# *What*, *where* and *when*: deconstructing memory

**DOI:** 10.1098/rspb.2013.2194

**Published:** 2013-12-07

**Authors:** Rachael E. S. Marshall, T. Andrew Hurly, Jenny Sturgeon, David M. Shuker, Susan D. Healy

**Affiliations:** 1 School of Psychology, University of St Andrews, St Andrews, UK; 2 School of Biology, University of St Andrews, St Andrews, UK; 3 Department of Biological Sciences, University of Lethbridge, Lethbridge, Alberta, Canada; 4 Institute of Evolutionary Biology, University of Edinburgh, Edinburgh, UK

**Keywords:** cognition, hummingbird, *what*–*where*–*when*, memory reconstruction, episodic-like memory

## Abstract

The ability of animals to remember the *what*, *where* and *when* of a unique past event is used as an animal equivalent to human episodic memory. We currently view episodic memory as reconstructive, with an event being remembered in the context in which it took place. Importantly, this means that the components of a *what*, *where*, *when* memory task should be dissociable (e.g. *what* would be remembered to a different degree than *when*). We tested this hypothesis by training hummingbirds to a memory task, where the location of a reward was specified according to colour (*what*), location (*where*), and order and time of day (*when*). Although hummingbirds remembered these three pieces of information together more often than expected, there was a hierarchy as to how they were remembered. *When* seemed to be the hardest to remember, while errors relating to *what* were more easily corrected. Furthermore, *when* appears to have been encoded as a combination of time of day and sequence information. As hummingbirds solved this task using reconstruction of different memory components (*what*, *where* and *when*), we suggest that similar deconstructive approaches may offer a useful way to compare episodic and episodic-like memories.

## 1. Introduction

Episodic memory is the system by which humans recall their past experiences and it is this experience that distinguishes it from semantic memory. While semantic memory covers ‘known’ memories, such as remembering that the Battle of Hastings took place in 1066, an episodic memory is one for which the individual has a sense of the event having occurred within a personal past [1], for example reminiscing about sitting in a classroom looking out of the window while learning about the Battle of Hastings. Given that the focus of this definition is on the individual's subjective experience, it has been suggested that episodic memory relies on faculties such as autonoetic consciousness (i.e. an ability to imagine ourselves in the past) and a sense of subjective time [2]. This renders episodic memory either a uniquely human ability or at least only (currently) accessible to study in humans.

Given this difficulty in testing episodic memory in animals, researchers have redefined the problem under the banner of ‘episodic-like’ memory [3]. This has been defined as the ability to integrate the *what*, the *where* and the *when* aspects of a unique past event and to use that memory flexibly to guide behaviour [4]. As a result of using this definition, the ability to act on these three components of a past event simultaneously has now been demonstrated in a range of species (including scrub jays, *Aphelocoma coerulescens*: [3], chickadees, *Poecile atricapillus*: [5], meadow voles, *Microtus pennsylvanicus*: [6] and rats *Rattus norvegicus*: [7]).

Although there is considerable debate regarding the extent to which episodic-like memory resembles episodic memory [8–10], the central tenet of episodic memory, the experience of the individual, is currently untestable and seems likely to remain so in the foreseeable future. There are, however, other features of episodic memory that are amenable to investigation within an episodic-like framework. For example, episodic memory in humans is regarded as reconstructive [11–13]: that is events are not stored as a whole in memory as by a video recorder, but rather via a system in which the elements of a memory are stored separately and recombined to create the event as it is recollected. This leads to various predictable errors when memory fails [14–16]. By contrast, episodic-like memory investigations typically require an animal to demonstrate that it can remember the three memory components together. If the same mechanisms underpin both episodic-like memory and episodic memory, we suggest that the components of memory of the former should also be dissociable and recombined at the point of recall. This reconstruction may on occasion be imperfect or incomplete, as is often the case for human memory, and thus the errors made in episodic-like memory tasks may be as informative as animals' successes. If animals make errors in episodic-like memory tasks that are comparable to the errors humans make when using episodic memory, perhaps these two systems are similar in form as well as function.

Hummingbirds provide a useful model system for studying the interplay of different sorts of information in memory, as they can remember numerous aspects of the flowers from which they feed, including their colour [17,18], location [19–22] and when they were last visited [23,24]: *what*, *where* and *when*. Here, we investigated whether in a system where the *what*, *where* and *when* components of memories can be experimentally manipulated, whether hummingbirds remembered all three pieces of information together as a whole or whether errors tended to reflect failure to recall one aspect of *what*, *where* and *when* (Experiment 1). If hummingbirds do not make errors at random with respect to *what*, *where* and *when*, then perhaps these pieces of information are stored separately in memory, in a manner consistent with the reconstructive model of episodic memory. In particular, we predicted that *when* would be the piece of information causing the most frequent errors, as this is frequently the most difficult aspect of episodic-like memory to demonstrate [25,26]. In our first experiment, birds might remember the temporal (*when*) component either by the sequence of rewarded flowers or by the time of day. In our second experiment, we tested whether the birds use time of day, sequence or a combination of the two.

## 2. Material and methods

### (a) Subjects

The subjects in these experiments were 18 free-living male rufous hummingbirds defending feeding territories along the Westcastle Valley, in the Eastern range of the Rocky Mountains (49°21′ N, 114°25′ W), Alberta, Canada (12 in Experiment 1; six in Experiment 2). Each territory was centred on a single hummingbird feeder, containing 14% sucrose solution. Birds were marked on their breast feathers with a small amount of non-toxic ink, to allow individuals to be identified. Observations were conducted between 07.30 and 19.30 (Mountain Standard Time). Experiment 1 was conducted in June–July 2005 and June 2006, and Experiment 2 from June to July 2008. All work was carried out under permit from Environment Canada and Alberta Sustainable Resource Development with the ethical approval of the University of Lethbridge Animal Welfare Committee.

### (b) Experiment 1

#### (i) Training

There were 2 days of training prior to the experimental procedure. On the first of these days, we presented each bird with an array of four flowers, coloured blue, red, pink or purple and arranged in a rough 60 × 60 cm square. One flower was filled with sucrose and the remaining three were filled with water, which the birds find unpalatable. The bird was allowed to visit this array until he had fed from the sucrose-filled flower six times, after which time the flowers were removed and the bird's feeder was replaced. This completed the training for the ‘Morning’ session. The ‘Afternoon’ training began at least 4 hours later. The bird was presented with the second array of four flowers of the same colours in the same relative positions but in a new location at least 10 m from the location of the morning array. For the afternoon array, the rewarded flower was not of the same colour as that rewarded in the morning array and the remaining three flowers contained water. Again, the bird was allowed to feed from the rewarded flower six times.

#### (ii) Experimental procedure

Following training, in the morning (at any time between 07.30 and 11.00, with the time broadly consistent across days for each bird), we removed a bird's feeder and presented him with both the morning and afternoon arrays of flowers simultaneously, arranged as they had been the previous day. The only flower of the eight to contain sucrose was the one that had been rewarded in the previous morning's training. All the remaining seven flowers contained water. The bird was allowed to visit any of the flowers until he had made six visits to the sucrose-filled flower, which was refilled after each visit, at which point both arrays were removed and the feeder was replaced. Four hours after both arrays were removed, they were returned but with the afternoon flower being the only flower containing reward. Again, the bird was allowed to visit flowers until he had visited the sucrose-filled flower six times. His feeder was then returned for the remainder of the day.

All visits to all flowers were recorded at both morning and afternoon sessions. The number of sessions (morning and afternoon included) experienced by each male varied from 5 to 17 (median = 12).

**Figure 1.**
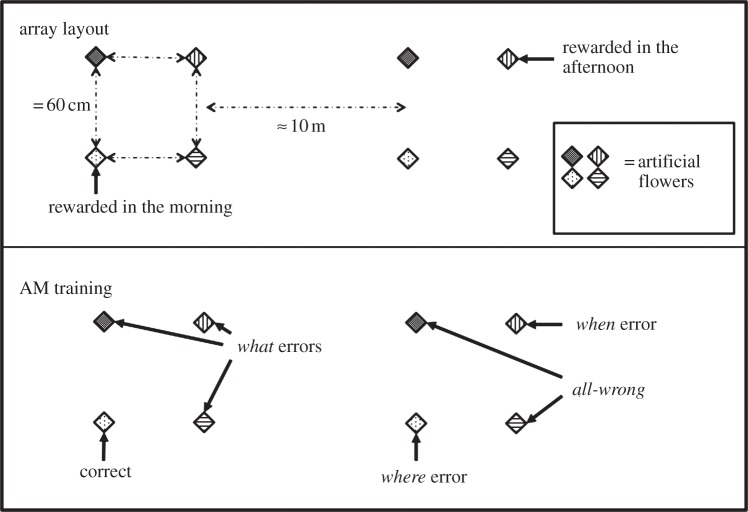
Schematic showing the two arrays of Experiment 1 and also the flower categorizations (e.g. *correct* or a *what* error) for morning trials.

In this design, there were three pieces of information that a bird needed to use in order to locate the rewarded flower: *What*: the flower's colour; *Where*: the array in which the flower is located and *When*: whether it is the morning or the afternoon*.* We could, therefore, look at whether these components of memory are separable by looking at the errors birds made, as they can be classified according to which of these pieces of information is missing from a bird's choice (figure 1). *What* errors were those where the bird chose the correct array at the correct time of day but a flower of the wrong colour (chance = 0.375); w*here* errors were those where the bird chose a flower of the correct colour at the correct time but in the wrong array (chance = 0.125); w*hen* errors were those where the bird chose the flower of the correct colour and in the correct array, but that was the flower that was rewarded at the alternative time (chance = 0.125); a*ll*-*wrong* errors were those where the bird chose a flower of the wrong colour, in the wrong place and at the wrong time (chance = 0.250). *Correct* choices were those in which the bird chose the rewarded flower (chance = 0.125).

There are two different aspects of birds' errors that we can examine in this experiment: the kinds of error made and how the birds went about correcting them. To determine whether some errors were easier to correct than the others, we looked at birds' subsequent choices after making an error, either up to the point where they located the rewarded flower or to their fourth choice within a trial (birds failed to find the rewarded flower within the first four visits on only 20 of a total of 134 trials). We then constructed a partition tree (R package rpart) modelling whether or not an error was corrected on a subsequent choice as a function of trial number, visit number within a trial, the type of error made on the last visit and the individual.

### (c) Experiment 2

In Experiment 1, *when* is defined both by time of day and sequence (i.e. the afternoon comes after the morning). To assess how birds were using these two components to estimate *when*, we conducted the second experiment in which we presented the birds with the same arrays as used in Experiment 1 in morning and afternoon sessions for 5 days. For Experiment 2, however, we included a test phase whereby birds were given an Early test: 2 hours before a morning session; a Midday test: in-between the morning and afternoon sessions; and a Late test: 2 hours after the afternoon session was completed.

Birds were allowed to make one visit to the arrays during each of these early/midday/late tests, after which all flowers were removed and the feeder was replaced. All flowers were empty during these tests. To reiterate, during testing birds continued to experience morning and afternoon trainings at the usual times and birds only experienced one test session per day. The order of the tests was randomized between birds and each bird completed each test only once.

Depending on whether birds used sequential or time of day information, or some combination of the two, birds' choices at these tests should differ and the explanations for how birds might choose a flower are as follows:

(i) Birds always chose randomly between the morning and afternoon flower, irrespective of time (*chance*). Prediction: birds would choose the morning and afternoon flowers equally across all three tests.
(ii) Birds always chose the morning flower, irrespective of time (*all AM*). Prediction: birds would choose the morning flower across all three tests.
(iii) Birds always chose the afternoon rewarded flower, irrespective of time (*all PM*). Prediction: birds would choose the afternoon flower across all three tests.
(iv) Birds always chose the flower corresponding to the time of day nearest to that to which they were trained (*time of day*). Prediction: birds would choose the morning flower in the early test, show no preference in the midday test and would choose the afternoon flower in the late test.
(v) Birds always chose the next flower due to be rewarded in the sequence (*sequence*: *avoid previous*). Prediction: birds would choose the morning flower in the early test, the afternoon flower in the midday test and the morning flower in the late test.
(vi) Birds always avoided the next flower due to be rewarded in the sequence (*sequence*: *avoid next*). Prediction: birds would choose the afternoon flower in the early test (as the next reward would be the morning flower), the morning flower in the midday test (as the next reward would be the afternoon flower) and the afternoon flower in the late test (as the next reward would be the morning flower the following day).
(vii) Birds combined sequential and time of day information to make their flower choices (*mixed*). Prediction: birds would choose the afternoon flower in the early test (as the morning flower would be rewarded at the time experienced during morning training and the rewarded flower alternates), equally between the morning and afternoon flowers in the midday test (as this test is midway between two known but different rewards) and the morning flower in the late test (as the last reward experienced was that of the flower from the afternoon and the reward alternates).

As the aim of these tests was to compare which of the two flowers (morning or afternoon) birds expected to contain the reward at untrained times of day, we modelled this test as a binary choice, where birds could either choose to visit the morning or the afternoon flower. This meant that we had to exclude cases in which birds made their first choice in a test to a flower that was never rewarded, which accounted for only one of 18 test trials. We used a likelihood approach to compare the likelihood of each hypothesis given the data [27]. We calculated the probability of observing the birds' choices under each of these scenarios and compared the negative log-likelihoods (−LL), with the smallest −LL denoting the most likely of the competing hypotheses [27].

## 3. Results

### (a) Experiment 1

**Figure 2.**
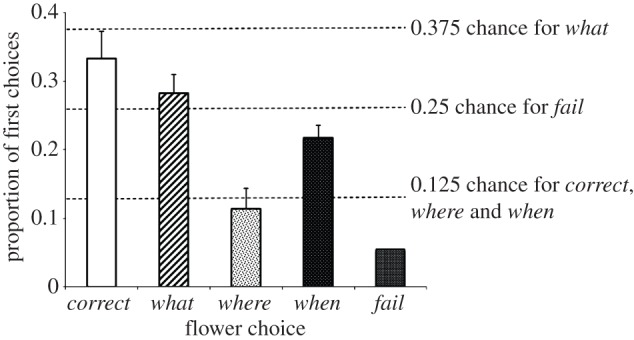
Proportion of first choices of each type in Experiment 1. The solid line at 0.125 represents chance for *when*, *where* and *correct* choices. The dashed line at 0.375 represents chance for *what* choices. The dotted line at 0.25 represents chance for *all*-*wrong* choices. Birds made correct choices significantly more often than chance, fewer all-wrong errors than chance, fewer *what* errors than chance and more *when* errors than chance.

Male rufous hummingbirds were able to learn a *what*, *where*, *when* task, making their first choice to the correct flower significantly more often than expected by chance (*t*_11_ = 6.16, *p* < 0.001). Correspondingly, birds made *all*-*wrong* errors significantly less often than expected by chance (*t*_11_ = 9.32*, p* < 0.001)*.* Critically, the prevalence of *what*, *where* and *when* errors differed. As predicted, *when* errors occurred more often than expected by chance (*t*_11_ = 3.09*, p* = 0.010)*.* Furthermore, *what* errors occurred less often than chance and *where* errors occurred at chance levels: (*what*: *t*_11_ = 2.41, *p* = 0.035, *where*: *t*_11_ = 0.71, *p* = 0.494; figure 2).

**Figure 3.**
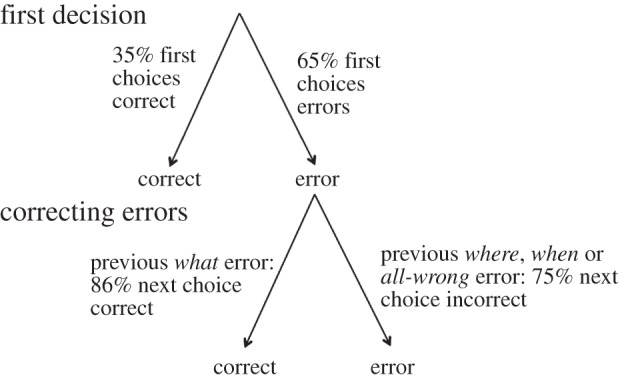
Hummingbird choices were corrected non-randomly. 47/134 choices of the birds’ first decision were correct, while 87/134 were errors. Where birds made *what* errors (whether on their first, second or third choice), 86% of the next choices were to the correct flower. However, if birds made a *where*, *when* or *all*-*wrong* error, 75% of the next choices were also wrong.

Not only were errors non-random, when males made a *what* error but also they were much more likely to visit the correct flower on their next choice (86% of choices following a *what* error) than when they had made a *where*, *when* or *all-wrong* error (25% of these errors; figure 3).

### (b) Experiment 2

**Figure 4.**
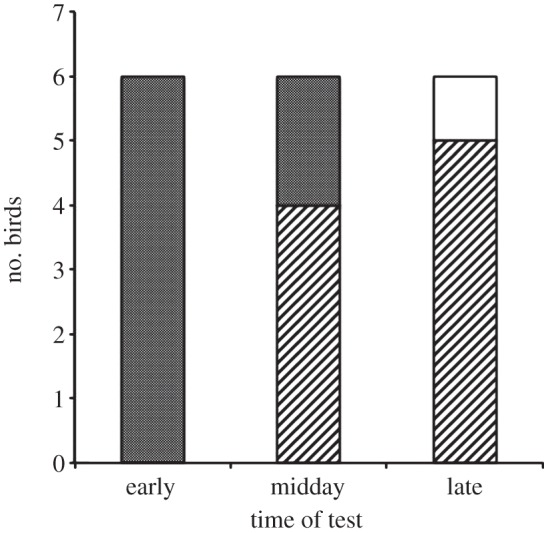
The number of birds that made their first choice to the morning and afternoon flowers at the early, midday and late tests. Striped portions of bars represent choices of the afternoon rewarded flower, clear bars represent choices of the morning rewarded flower and dotted bars represent choices of unrewarded flowers.

In early tests, the birds' chose the flower rewarded in the afternoon significantly more often than expected by chance: all six birds chose the afternoon rewarded flower (two-tailed test binomial test: *N* = 6, test-proportion = 0.5, exact *p* = 0.031; figure 4). In the midday tests, the birds' performance did not differ significantly from chance: four birds chose the flower rewarded in the morning and two chose the flower rewarded in the afternoon (two-tailed binomial test: *N* = 6, test-proportion = 0.5, exact *p* = 0.688). In the late tests, birds tended to choose the flower rewarded in the morning (two-tailed test binomial test: *N* = 5, test-proportion = 0.5, exact *p* = 0.063): five birds chose the flower rewarded in the morning while the sixth bird chose a flower that was never rewarded (we excluded this choice from the analysis). When we compared the birds' performance across the early, midday and late tests with our hypotheses, their choices were most consistent with those predicted by the mixed hypothesis (−LL = 0.678; table 1).

**Table 1.** A likelihood analysis of possible decision rules that male hummingbirds may have used to choose which flowers to visit in Experiment 2. We tested seven hypotheses, including random choice (chance) and fixed choice rules (all morning flowers or all afternoon flowers) plus four hypotheses related to time of day, sequence and a combination of time and sequence (see main text for more details). The negative log-likelihoods (–LL) of obtaining the observed patterns of flower choice across the three tests (early, middle and late) under each hypothesis were calculated using the binomial probability distribution. The smallest value of –LL indicates the hypothesis with the best support given the data. When a hypothesis involves the probability of choosing the morning flower being either *p* = 1 or *p* = 0 (i.e. always choosing the morning flower or never choosing the morning flower), we used *p* = 0.99 and *p* = 0.01 to facilitate the likelihood calculations.

hypothesis	expected probability of choosing the morning flower		negative log-likelihood (−LL)	
	early	midday	late	sum
(1) chance	0.50	0.50	0.50	3.94
(2) all AM	0.99	0.99	0.99	14.90
(3) all PM	0.01	0.01	0.01	16.90
(4) time of day	0.99	0.50	0.01	22.60
(5) sequence (avoid previous)	0.99	0.01	0.99	18.90
(6) sequence (avoid next)	0.01	0.99	0.01	12.90
(7) mixed	0.01	0.50	0.99	0.68

## 4. Discussion

Rufous hummingbirds' errors on this *what*, *where*, *when* task were not random. Errors very rarely represented a failure to remember any aspect of the rewarded flower (i.e. *all*-*wrong* errors), rather their errors generally represented the failure to remember one aspect of the *what*, *where* or *when*. In particular, birds were most likely to make errors regarding *when* a flower should be rewarded. This is consistent with other studies, where the time component appears to be the most difficult for many animals to learn [25,26]. Furthermore, birds made *what* errors less frequently than expected by chance. It therefore appears that these birds store *what*, *where* and *when* as separate pieces of information, as is thought to be the case for human episodic memory [13].

Not only were birds less likely to make *what* errors, when these errors did occur they were more likely to be followed by a visit to the correct flower. Here, *what* is signalled by flower colour, and previous research has suggested that while hummingbirds can use colour to direct foraging when no other cue is available [17,18], colour information tends to be overshadowed by spatial information [22,24,28]. The ready correction of *what* errors would suggest that birds knew the correct flower colour but preferred to use other information first. It is worth noting that not all *what* errors in this experiment were equivalent: birds either chose the flower colour rewarded at the alternative time (one of three flowers) or a flower colour that was never rewarded (two of three flowers). It seems that there is a difference in how readily birds made these two types of errors: most of the birds' *what* errors in training were to the colour rewarded at the other time (33 of 37 initial w*hat* errors), rather than to one of the two never rewarded colours. Given that most of these *what* errors were to a specific type of flower, the proportion of *what* errors relative to chance was actually very high (figures 2 and 3). It seems likely that birds may have learned which flower colours were rewarded and which were not, but their errors stemmed from failing to integrate this information with *where* and *when*. We cannot, however, yet exclude the possibility that owing to the distance (10 m) that separated the two arrays correcting *what* errors was less costly than was correcting *where* or *when* errors. Testing this possibility (and indeed exploring the optimal use of multi-component memory more generally) requires further experimentation.

Based on the difficulty that animals often have had in remembering the *when* component in episodic-like experiments [25,26], we predicted that the birds would find the *when* component of the task the most difficult to remember, which was indeed the case. However, this component of the task was distinct from the *where* or *what* components in that there was more than one kind of information about when that the birds could have used, as the rewarded flower could have been predicted by both the time of day and by the sequence in which arrays were rewarded across the day. Experiment 2 suggests that the hummingbirds used both time of day and sequence information in combination: they learned a sequence (i.e. that the reward in the two flowers alternated) but they also learned that the sequence was anchored to specific times of day. This explanation is consistent with the finding that rats can combine different types of information to guide their behaviour [29].

Under its original formulation, animals must remember all three of *what*, *where* and *when* simultaneously in order to show episodic-like memory [4]. However, this all-or-nothing approach to memory is not in close accord with human episodic memories, which are often incomplete and thought to be recalled by using reconstructive processes, where an episode is reassembled as it is recalled [12]. While our experiment does not directly test episodic-like memory, as animals received repeated trials on the same task, our data suggest that the *what*, *where* and *when* components of a memory may likewise be separable in animals. The separability of these components of a memory is supported by the variation in memory impairments in mice trained to a *what*, *where* and *when* task [30]: hippocampal-lesioned animals were impaired on the overall task, while mice with lesions to the prefrontal cortex were specifically impaired on the *where* element of the task.

If *what*, *where* and *when* are indeed remembered in a reconstructive way by hummingbirds and other animals, there may be a number of ways to investigate the similarities (or not) between human episodic memory and animal episodic-like memory. In humans, the integrative nature of episodic memory leads to a variety of memory failures that may be amenable to testing in animals. These include generalization, where subjects incorrectly remember aspects of an event that did not occur owing to their close relation to an event's actual context. For example, subjects will frequently ‘remember’ words they have not seen if a list of words contains other thematically related words, such as remembering *hospital* having seen the words *ambulance*, *doctor*, *operation*, *X-ray*, *ward*, etc*.* [14,15]. Another type of reconstructive memory error is blending, where two memories which share many features can be confused with each other and combined to make a novel (and inaccurate) memory [16]. Given that our experiment suggests that information about *what*, *where* and *when* are also separable in animal memory, it seems to us that it would be useful to look for these kinds of reconstruction errors in episodic-like tasks, to see whether animal memory resembles human memory in form as well as content.
